# Global calling to develop geroscience research and promote healthy aging—Key discussion points from the International Geroscience Symposium in Shenzhen, China

**DOI:** 10.1002/agm2.12081

**Published:** 2019-10-06

**Authors:** Yiyin Chen, Sean X. Leng

**Affiliations:** ^1^ Guangdong Geriatrics Institute Guangdong Provincial People's Hospital & Guangdong Academy of Medical Sciences Guangzhou China; ^2^ Division of Geriatric Medicine and Gerontology Department of Medicine Johns Hopkins University School of Medicine Baltimore MD USA

## 1 INTRODUCTION

With the support from the National Institute on Aging (NA) Nathan Shock Centers and Milstein Medical Asian American Partnership (MMAAP) Foundation and in collaboration with the American Federation for Aging Research (AFAR) as well as Chinese Medical Association Geriatrics Branch and China National Center on Geriatrics and Gerontology, an International Geroscience Symposium, the first of a series of seven geroscience conferences worldwide, was successfully held on May 24‐25. 2019, at Marco Polo Hotel in Shenzhen, China. This was held in conjunction with the 2019 annual scientific conference of Chinese Medical Association Geriatrics Branch. The symposium mainly covered the following seven scientific areas in focus: (a) Principles of geroscience; (b) Pillars of aging, resilience, and immune aging; (c) Anti‐aging research and translation; (d) Traditional Chinese Medicine and aging; (e) Stem cell research and regenerative medicine; (f) Artificial intelligence and aging; and (g) Infrastructure and platform building for geroscience. Over 40 speakers from inside China as well as around world with expertise in aging and related research fields participated and presented at the Symposium. In addition to a 45‐minute discussion scheduled at the end of each scientific session, there was a closed executive session among the speakers at the end of the Symposium to discuss the state of the field as well as ways and strategies to further promote and build geroscience both in China, Southeast Asia and around the world. The following is a brief summary of key points from a number of leading participants in this session.

## 2 KEY DISCUSSION POINTS

The primary objective of geroscience is to promote healthy aging rather than to treat diseases. As such, the scope of geroscience includes the deciphering of mechanisms that underlie aging, the most significant risk factor for many, if not all, diseases. Strategies to prevent and intervene on diseases and disability can be developed for the aging population. Similar to other research areas of biology, geroscience can utilize all kinds of animal models. For example, we can study the natural history and pathology of Alzheimer's disease using the brain of an aging mouse. One aspect of the real impact that geroscience can have is to make older people more active and engaging rather than merely healthier. If an older person only sits in the room watching TV, his or her health, psychological well‐being, and social interaction are probably not good. S/he would miss a huge opportunity to apply his/her wisdom, skills, and experiences to contribute to the society as well. A concept of the fourth age life where we re‐engage older individuals after retirement, train them for new skills and keep them active has developed. Similarly, re‐engaging them to create a third demographic dividend not only enables healthy aging, but also strengthen intergenerational well‐being in ways that deeply matter.

Normally, geriatricians screen patients in many aspects to find evidences of a disease. Instead of this disease‐oriented approach, however, a solid, strong and realistic age‐oriented approach is needed to be implemented in the near future, and there is a need to educate physicians, researchers and other thinkers on the concept that an age‐oriented approach is more helpful to promote health. What we need to do is to collect the evidence supportive of benefits from this age‐oriented approach in both animal models and human studies. For example, exercise is proven to be an effective intervention on aging, and substantial evidence has shown that exercise improves health via a number of pathways. With growing evidence supporting the great beneficial impact derived from an age‐oriented approach, scientific communities, politicians as well as government and funding agencies may realize that this is a different, better and more efficient approach.

Geroscience is at different development stages in different countries. It is growing gradually, but it is still at its infancy in countries like Australia and China. We should make such countries realize the benefits of promoting geroscience and help them make progress. So how to promote geroscience in countries like China? First, we should try to raise more funds which consistently support research in geroscience. The branch of geroscience has been funded by the National Institute on Aging (NIA) in the US for years. We hope that in the future geroscience will become a focused and priority area of research funding in China, adequately funded by the National Natural Science Foundation and other government funding agencies as well as local and international foundations (eg, Milstein Medical Asian American Partnership Foundation, http://www.mmaapf.org). Secondly, it is necessary to publish scientific articles introducing geroscience in local journals so that more people might have a better understanding of geroscience. Third, as the government plays a very important role in policy making, the concept of geroscience needs to reach governmental spheres, so as to facilitate the wider promotion of geroscience. As far as we know, government agencies fund research on many things including social science, policy research, clinical geriatrics, etc. It is worthwhile trying to convince government officials and agencies about the importance of geroscience, and convince government officials to play a role in promoting it. Last, as China has very few scientists with a focus on aging research at the present time, we should train more scientists in aging research who, in turn, will be qualified to train the next generation of aging researchers.

Many people think that longer lives mean an unsustainable increase in healthcare costs. Thus, we recommend the conduct of a comprehensive economic impact analysis of extending health span by 1, 2, or 10 years. Such analysis would be very valuable for the government and helpful to let people realize the value of geroscience. America and Singapore have been doing similar analyses now. In Taiwan, geroscience is a research area of high quality and is getting more support from scientific communities. However, the economic impact analysis has not yet been done. In mainland China, results of such analysis were presented with great attention at a conference in Hainan Island. Researchers in the Academy of Social Science have also done similar analyses. In the future, it would be helpful if senior economists could cooperate with biomedical scientists working on geroscience for such work. The depth of the analysis will be influenced by the amount of funds, so the full cost of the analysis should also be analyzed.

The Geroscience Conference in China is the first one from a set of seven similar conferences. Each conference is expected to have a slightly different “flavor,” dictated by the realities of each geographic area. A special feature or “flavor” of this symposium is traditional Chinese medicine (TCM) which is unique to China. TCM, with its ancient history, focuses on holistic health rather specific diseases. While quite in contrary to the disease‐focused dogma of Western medicine, TCM echoes well with geroscience which focuses on healthy aging. This international symposium in China has attracted many researchers in aging and spread the basic concept of geroscience. Taken together, it is expected that these geroscience conferences will result in a unified message, and issue an important discussion document, including a very clear definition of the term “geroscience.” In this way, people can understand the concept of geroscience better. An important feature of all the conferences is that they are suggested to be guided, rather than managed, thus allowing for open discussions and rather unrestricted conversations.

In this international geroscience symposium, we introduced the principles of geroscience, pillars of resilience and immune aging, anti‐aging research and translation, traditional Chinese medicine and aging, stem cell research and regenerative medicine, artificial intelligence and aging, infrastructure and platform building for geroscience. It is necessary for us to make a statement of our discussion to let more people know about our work. First, we should publish scientific papers on research using age‐oriented approaches. Secondly, we should discuss with editors of high impact academic journals to develop special issues publishing on geroscience. While there is a journal named *Geroscience*, special issues in non‐aging journals will enable our message to reach broader readership. Social media is another way which could reach out to even more people. In addition, we should generate more discussion on aging and geroscience, not only as a scientific research papers, but also as viewpoints and editorials. Things like a viewpoint or sounding board about geroscience could express our opinion about geroscience as a group. What we expect to do is to make geroscience claims backed up by solid existing evidence. Thus, it should be a combination of reviews and viewpoints. We recommend that scientists working in basic medicine in East Asia start preparing such a manuscript.

## 4 CONFLICTS OF INTEREST

Nothing to disclose.

